# Spatial grain of adaptation is much finer than ecoregional‐scale common gardens reveal

**DOI:** 10.1002/ece3.6651

**Published:** 2020-08-19

**Authors:** Bill E. Davidson, Matthew J. Germino

**Affiliations:** ^1^ Forest and Rangeland Ecosystem Science Center U.S. Geological Survey Boise ID USA

**Keywords:** adaptive variability, common gardens, native plant establishment, seed zones

## Abstract

Adaptive variation among plant populations must be known for effective conservation and restoration of imperiled species and predicting their responses to a changing climate. Common‐garden experiments, in which plants sourced from geographically distant populations are grown together such that genetic differences may be expressed, have provided much insight on adaptive variation. Common‐garden experiments also form the foundation for climate‐based seed‐transfer guidelines. However, the spatial scale at which population differentiation occurs is rarely addressed, leaving a critical information gap for parameterizing seed‐transfer guidelines and assessing species’ climate vulnerability. We asked whether adaptation was evident among populations of a foundational perennial within a single “empirical” seed‐transfer zone (based on previous common‐garden findings evaluating very distant populations) but different “provisional” seed zones (groupings of areas of similar climate and are not parameterized from common‐garden data). Seedlings from three populations originating from similar conditions within an intermediate elevation were planted into gardens nearby at the same elevation, or 250–450 m higher or lower in elevation and 0.4–25 km away. Substantial variation was observed between gardens in survival (ranging 2%–99%), foliar crown volume (7.8–22.6 dm^3^), and reproductive effort (0%–65%), but not among the three transplanted populations. The between garden variation was inversely related to climatic differences between the gardens and seed‐source populations, specifically the site differences in maximum–minimum annual temperatures. Results suggest that substantial site‐specificity in adaptation can occur at finer scales than is accounted for in empirical seed‐transfer guidance when the guidance is derived from broadscale common‐garden studies. Being within the same empirical seed zone, geographic unit, and even within 10 km distance may not qualify as “local” in the context of seed transfer. Moving forward, designing common‐garden experiments so that they allow for testing the scale of adaptation will help in translating the resulting seed‐transfer guidance to restoration projects.

## Graphical abstract

Seed‐transfer guidelines form an important means for translating our knowledge about climate adaptation that is derived from common‐garden studies into restoration of plant communities. We show that seed‐transfer guidelines cannot predict the survival, growth, or reproduction of plants transplanted within a restoration landscape, likely due to the coarse‐scale parameterization of the gardens and resulting guidelines. Moving forward, incorporating a hierarchical blend of different scales of climate and geographic separation of populations used in the common gardens and guidelines should increase their applicability to seed transfer within landscapes.

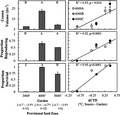

## 1 INTRODUCTION

Adaptive variability among populations that results from geographic, genetic, or environmental isolation is key to understanding species’ responses to stressors such as climate shifts and to species’ conservation and restoration (Rodríguez‐Quilón et al., 2016). Of particular importance is site‐specific adaptation which can often be referred to as "local" adaptation, i.e. superior fitness of a population within its geographic origin compared to other populations within the same taxonomic group, evident as a “home‐site” advantage for local populations (Bennington et al., 2012; Bucharova et al., 2017; Johnson et al., 2015; Joshi et al., 2001; Montalvo & Ellstrand, 2001b). However, “site‐specific” or “local” is vague and quantitative definitions of them are rare, and thus the terms are difficult to implement for research and management applications. The spatial scale and degree of site‐specific or local adaptation is unknown for many foundational native species, and moreover likely varies considerably among species as a function of breeding system, gene flow and its relationships to population size, dispersal, and landscape heterogeneity (De Carvalho et al., 2010; Forester, Jones, Joost, Landguth, & Lasky, 2016; Lenormand, 2002; McKay, Christian, Harrison, & Rice, 2005; Stacy et al., 1996). Meta‐analyses of local adaptation across a wide range of annual and perennial plant taxa found evidence of local adaptation in many but not all studies (Hereford, 2009; Leimu & Fischer, 2008). Geographic or environmental distance of populations was not considered in these meta‐analyses and could account for much of the variability among studies.

Common‐garden studies have been the primary means of detecting adaptive differentiation in plants and the resulting information has been used to parameterize seed‐transfer guidelines. The studies compare survival, growth, and other traits of populations collected from different areas and then planted together (Castellanos‐Acuña et al., 2018; O’Neill, Stoehr, & Jaquish, 2014). Analysis of common gardens can include the “genecological” approach, in which genetic variation among the co‐planted populations is related to conditions of their origins, such as climate. Studies using this approach with trees and widespread grasses differ in whether substantial adaptive variation was evident (e.g., Durka et al., 2017; De Kort et al., 2014; Bradley St. Clair, Kilkenny, Johnson, Shaw, & Weaver, 2013). These studies tend to either evaluate (a) broadscale, ecoregion‐wide population variability with source populations from across the species’ range, or (b) fine‐scale variability that can be attributed to subspecific or other subtaxonomic variants that are recognized a priori across environmental gradients. The distance between source populations in broadscale gardens typically entail hundreds to thousands of km of separation. The lack of variability in transfer distances, that is, combining both broad and fine‐scale population interdistances, leads to uncertainty in the spatial grain of any adaptive variation observed in the common garden. Two key, unanswered questions are sources of substantial uncertainty in applying climate‐based seed zones or conducting climate vulnerability analyses. These questions are (a) how site‐specific is adaptation? and (b) can seed‐transfer guidelines be reliably applied within landscapes, even large ones? These questions can be addressed by validating empirical seed‐transfer guidelines with common gardens that are established independently from the gardens that are or were used to parameterize the seed‐transfer guidelines.

These questions are highly relevant because the default seed‐selection strategy where empirical seed‐transfer guidelines are not available is to use the most local seed sources available (Boshier & Stewart, 2005; McKay et al., 2005). Undisturbed, remnant stands within or along the periphery of these large restoration areas serve as seed sources to the surrounding landscape, either passively through natural seed dispersal, or through active seed collection and redistribution (planting) by restorationists, often across 10s to 100s of km. Land management agencies which use large quantities of seeds annually typically rely on collections made from even more distant populations, for example, 300–600 km, and from different climate gradients (Germino, 2014). Such seed transfers have the potential to introduce maladapted seed sources that can reduce establishment success, alter the gene pool of surrounding populations, influence adaptation to climate and other stressors, and alter the ecosystem functioning of species, including palatability and/ or suitability of the habitat for wildlife (Montalvo & Ellstrand, 2001a; Pedlar et al., 2012; Potter & Hargrove, 2012; but see Tigano & Friesen, 2016) such as a potential phenological mismatch between plants and pollinators (Straka & Starzomski, 2014). Small‐scale seed transfer from unburned remnants to other sites within larger disturbance areas is increasingly common, particularly in the production of nursery seedlings for outplanting. The increase in disturbance frequency and extent has made these local collections from remnant stands less feasible as well as lost genetic diversity as a result of habitat loss and climate change (Breed, Stead, Ottewell, Gardner, & Lowe, 2013). Wildfires and other landscape disturbances encompass increasingly large areas of 50,000 to 500,000 ha with substantial environmental gradients and thus likely contain variable selection pressures that can result in the development of adaptive variation among the species being restored within project boundaries.

We asked how much adaptive variation exists within a landscape for a single subspecies of big sagebrush, *Artemisia tridentata* subsp. *wyomingensis,* hereafter, "big sagebrush". Big sagebrush is one of the most broadly distributed and locally abundant foundational species in North America yet is also imperiled (Miller et al., 2011) and has been among the most extensively seeded wildland species, worldwide. Sagebrush ecosystems have experienced substantial degradation due to exotic grass invasion and increases in wildfire size and frequency, which the species is poorly adapted to owing to its inability to resprout or form enduring seed banks compounded by its slow maturation and frequent fire recurrence. Approximately half of the original 1,000,000 km^2^ of sagebrush steppe that once existed has been converted to annual grasses in recent decades, and so restoration of the species is attempted over large areas. Aerial broadcast seeding or seedling plantings of sagebrush are often met with mixed results (Arkle et al., 2014; Davidson, Germino, Richardson, & Barnard, 2019; Knutson et al., 2014), possibly owing to a lack of selectivity in seed sources used (Oldfield & Olwell, 2015). Sagebrush seeding began in the late 1980s and has been aerially broadcast on millions of hectares on hundreds of burned areas (Pilliod, Welty, & Toevs, 2017) since then. Sagebrush seed is collected from wild populations, and while very large collections are needed to supply the vast areas seeded, use of seed zone guidance did not occur until 2015, when provisional seed zone map derived from ecoregional classification and climatic conditions (Bower, St Clair, & Erickson, 2014) became available. Empirical seed‐transfer guidelines specific to big sagebrush that were based primarily upon phenology and survival data collected from three large‐scale common gardens (Richardson & Chaney, 2018) later became available and are in use as of 2020. Sagebrush are also increasingly reared in nurseries and then outplanted into burned areas in hopes of having higher establishment success (e.g., Davidson et al., 2019), in much smaller quantities which enable using “local” seed sources. Thus, seed selection can either be from local or distant sources, and either way needs to be guided by selection criteria.

We compared the survival, growth, and reproductive effort of three intermediate elevation populations of Wyoming big sagebrush planted in gardens along an elevational gradient over three growing seasons. We hypothesized that fitness (survival, reproduction, growth) would be greatest in the garden that had climatic conditions most similar to the seed‐source origins (i.e., indicating greater site‐specific and possibly local adaptation). We then compared our findings to published seed‐transfer guidelines, which includes (a) Bower et al.'s (2014) provisional seed zone map and (b) Richardson and Chaney's (2018) empirical seed‐transfer guidelines.

## 2 MATERIALS AND METHODS

### 2.1 Seed collection

**FIGURE 1:**
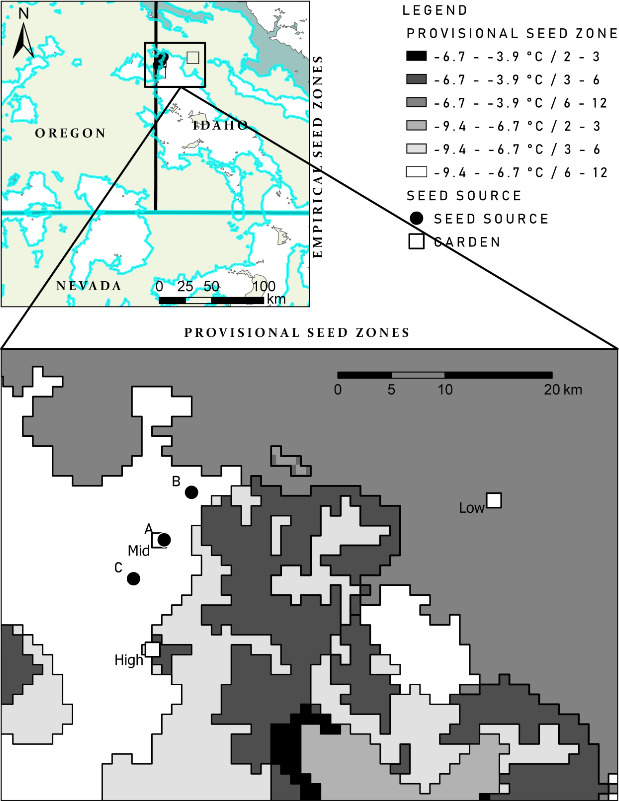
Map of seed source (round symbols) and garden (squares) locations in the Owyhee Mountains, Idaho. Top left panel: Empirical Seed zone (Chaney et al., 2017), garden and seed‐source locations. Lower panel: Seed source and garden locations in relation to Provisional Seed Zone delineation based upon 2.8°C bands of minimum winter temperatures (December through February) combined with groupings of the annual heat: moisture index (AH:M), a measure of aridity [mean annual temperature (°C) divided by mean annual precipitation (m, Bower et al., 2014)]

Seeds were collected from isolated, remnant, unburnt patches of Wyoming big sagebrush within the Soda wildfire perimeter (burned in 2015) that were separated by 4–5 km, and each population was randomly assigned a unique identifier: “A,” “B,” or “C.” Each population collection pooled seed from 70 to 100 individuals. Seed source “A” was collected from 1,341 m above sea level (43.30°N, −116.99°W), seed source “B” was collected from 1,312 MASL (43.34°N, −116.96°W), and seed source “C” was collected from 1,263 MASL (43.27°N, −117.01°W, Figure 1). The source populations and plants used had 100% trait fidelity to *Artemisia tridentata* subsp. *wyomingensis* in crown and leaf morphology, leaf chemistry, and cytotype. The lateral, geographic transfer distance (difference between planting site and seed source) ranged from 0.4–25.8 km, and the change in elevation ranged from −455 to 313 m (Table 1). Seed collections were cleaned, sown, and reared according to standard protocols at the U.S. Forest Service Lucky Peak Nursery (approximately 100 km to the east, 43.581°N, −115.990°W, Fleege, 2010).

**TABLE 1:** Vertical differences (elevation, m) and lateral distances (km) between seed‐source locations and the gardens (designated in ft)

Garden (relative elevation)	Seed collections from mid‐elevation		
	A	B	C
Low			
Elevation difference (m)	−451	−455	−387
Lateral distance (km)	22.7	20.6	25.8
Mid			
Elevation difference (m)	3	−1	67
Lateral distance (km)	0.4	5	4
High			
Elevation difference (m)	248	244	313
Lateral distance (km)	10.3	15	6.8

Note

Negative numbers indicate that the garden is at a lower elevation than the seed‐source location.

### 2.2 Garden preparation and planting

Garden sites were established near 914 (“Low”), 1,219 (“Mid’), and 1,524 MASL (“High”) in elevation within the Owyhee Mountains, Idaho, United States (Figure 1). Garden locations were tilled and treated with pre‐emergent herbicide (imazapic at a rate of 6 oz./acre = 420.32 g/ha). Seedlings generated from seed were grown for 6 months and hardened outdoors for 3 weeks prior to planting in mid‐November 2016. In each garden, 20–50 plants from each of the three populations/seed sources were randomly distributed into a planting grid with 1.5 m spacing. Seedlings were watered at the time of planting and once per week for 2 weeks thereafter soils were persistently wet from winter precipitation.

### 2.3 Monitoring

Our analyses focused on survival, growth, and reproduction, which are primary indicators of plant fitness. Plant survival (Alive or Dead) and crown volume was assessed at each garden from 9 to 13 times during the following 3 years. Crown volume (cm^3^) was determined from measurements of plant height as well as greatest and perpendicular crown widths (to 0.5 cm resolution) and calculated by assuming crown shape was an oblate spheroid [Volume = (4/3)*πabc*], where the radii of *a* and *b* are crown width and *c* is shrub height. Reproductive effort was quantified as the number of individuals within a population producing reproductive stalks in the fall of 2018.

### 2.4 Climate and weather

Gridded, 30‐year climate averages (1980–2010) were acquired from PRISM Climate Group (2015) and used to calculate climate variables, summarized in Table 2. To compare weather conditions to averaged climate variables, weather data were also acquired from PRISM Climate Group (2015) for the reported study period (November 2016–May 2019, Figure 2). In all, eleven climate variables were calculated relating to temperature averages and extremes, precipitation amount and timing, aridity, as well as annual degree‐day accumulation greater than 5°C and less than 0°C. The difference in climate variable values between source and garden locations (“climate transfer distance”, or CTD) were evaluated to identify factors most strongly correlated with seedling fitness variables (survival, growth, and reproduction).

**TABLE 2:** Elevation and climate variables of garden and seed source (A, B, C) locations

Garden/source	Elevation (m)	Soil type	Soil subgroup	MAT (°C)	MAP (mm)	DD > 5	DD < 0	MTCM (°C)	ADI	CTD	SMRP	SMRPB	SPRP	WINP
High	1,570	Loam	Argiduridic Durixerolls	8.12	362.1	1,813.7	356.5	−2.31	0.12	22.8	20.16	0.33	74.4	154.3
Mid	1,285	Loam	Abruptic Xeric Argidurids	8.20	322.1	1,805.6	336.9	−2.06	0.14	22.3	20.21	0.35	71.3	129.5
Low	885	Sandy‐Loam	Xeric Haplargids	10.42	246.5	2,260.6	221.0	−0.89	0.21	23.6	15.31	0.38	52.2	102.0
A	1,341	Loam	Abruptic Xeric Argidurids	8.00	325.4	1,781.2	359.5	−2.38	0.14	22.5	21.32	0.37	70.5	127.2
B	1,312	Loam	Xerollic Paleargids	8.21	335.1	1,830.1	350.3	−2.23	0.13	22.7	21.36	0.36	71.6	131.5
C	1,263	Loam	Abruptic Xeric Argidurids	8.21	327.4	1,823.8	347.5	−2.27	0.14	22.7	21.18	0.35	72.1	129.2

Note

Mean annual temperature (MAT, °C), mean annual precipitation (MAP, mm), degree days greater than 5°C (DD > 5), degree days less than 0°C (DD < 0), mean temperature of the coldest month (MTCM, °C), annual drought index (ADI, unitless), climatic temperature difference (CTD, °C, difference between mean temperature of the warmest month and mean of the coldest month), summer precipitation (SMRP, mm, July and August), summer precipitation balance (SMRPB, unitless, summer precipitation/spring precipitation), spring precipitation (SPRP, mm, April and May), winter precipitation (WINP, mm, November to February).

**FIGURE 2:**
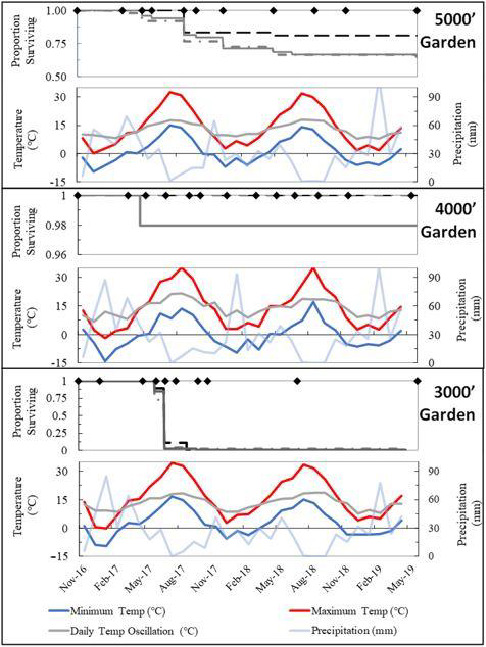
Timing and extent of mortality events in context of weather conditions during the reported period for the 5,000ʹ, 4,000ʹ, and 3,000ʹ gardens. Kaplan–Meier survival curves for each population (black dashed is 4000A, gray dashed is 4000B, solid gray is 4000C). Temperature (°C, minimum and maximum) and daily temperature oscillation (°C, difference between monthly mean maximum and minimum temperature), and cumulative monthly Precipitation (mm). Black diamonds indicate monitoring events for each garden

### 2.5 Seed zones and soil taxonomy

Published provisional (Bower et al., 2014) and empirical (Richardson & Chaney, 2018) seed zone shapefiles were downloaded from the USDA Forest Service Western Wildland Environmental Threat Assessment website (https://www.fs.fed.us/wwetac/). Soil taxonomy of seed collection and garden sites were identified to subgroup (SSURGO, Soil Survey Staff). Soil texture was estimated from field‐collected soils characterized using the feel method (Thien, 1979). Spatial data were mapped in a geographic information system (ArcGISPro 2.3.0; ESRI Corporation, Redlands, California, USA 2018).

### 2.6 Analysis

Collected data on seedling fitness metrics (growth, survival, and reproductive effort) were analyzed to identify differences between populations and gardens and characterize the relationships between population fitness metrics to the abiotic conditions of the gardens, relative to the conditions of the seed‐source site. We did not expect differences among the populations because of the similar, intermediate conditions from which we obtained their seed, but we nonetheless sought to confirm our assumption in the statistical models.

The proportion of both surviving and reproducing individuals as well as crown volume were compared between populations and gardens using type 3 ANOVAs, due to unequal sample sizes, using the car package (Fox & Weisberg, 2019). Means comparisons were conducted using the Tukey HSD. Each climate variable was tested in a separate model in order to maintain enough degrees of freedom. We did not use a multivariate derivative combination of variables because we wished to know the importance of specific climate variables that relate to different mechanisms by which climate affects sagebrush. The relative importance of garden, population, climate distance variables, and their interactions in explaining the variability in fitness metrics were evaluated using stepwise linear mixed model fit by restricted maximum likelihood (REML) with the packages lme4 (Bates, Maechler, Bolker, & Walker, 2015) and lmtest (Zeileis & Hothorn, 2002). The corresponding climate distance variables for each garden were treated as fixed effects. Population identity and interactions involving population identity were included as a random effect, and analyses were weighted by the number of seedlings within each population per garden. Several climate distance variables were correlated including CTD, SMRP, SMRPB, and SPRP with elevation (m) as well as CTD with degree‐day variables so separate models were tested for each factor. Climate transfer distance values were unique for each combination of population and garden; hence, each was treated as a separate sample for regressions. Differences in survival rates and timing of mortality were evaluated using Kaplan–Meier analysis using the survival package (Therneau, 2015). Kaplan–Meier analysis evaluates time‐to‐mortality using a nonparametric approach and allows comparison of mortality rates between populations or groups. All analyses were conducted in Rstudio (R Core Team, 2018).

Although our experiment could test for site‐specificity in adaptive variation, it did not include reciprocal plantings and thus could not provide the most formal, rigorous test for local adaptation (Blanquart, Kaltz, Nuismer, & Gandon, 2013). However, the “home versus away” approach we used can provide strong evidence for the imprint of natural selection, consistent with evidence for local adaptation (Blanquart et al., 2013; Kawecki & Ebert, 2004).

## 3 RESULTS

### 3.1 Seedling fitness

**FIGURE 3:**
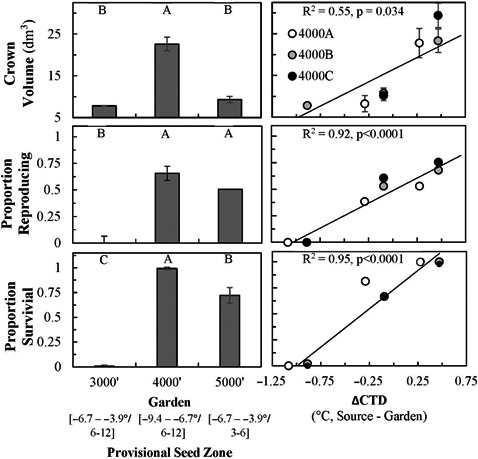
Differences in survival, reproduction, and growth between garden and provisional seed zones, and their correlation with the change in Climatic Temperature Difference (°C, ΔCTD = ΔT_source_–ΔT_garden_). (1) Crown volume (dm^3^), (2) proportion of reproducing individuals and (3) proportion of surviving seedlings. Error bars depict standard error (±*SE*). Different letters indicate statistical significance between gardens (*p* < .05). Provisional seed‐ transfer zones (Bower et al., 2014) delineated by minimum winter temperatures (°C) and annual aridity index (AH:M, °C/m)

Survival (*R* ^2^ = .99), crown volume (dm^3^, *R* ^2^ = .89), and the proportion of reproducing plants (*R* ^2^ = .91) differed significantly between gardens but not among populations within each garden (Table 3). Survival, crown volume, and reproductive effort were all substantially greater at the Mid garden, least in the Low garden, and intermediate in the High garden (Figure 3). Stepwise standard least squares regression identified that the most significant variable explaining seedling survival was the difference in continentality of climate between the population origin and each garden, calculated as ΔCTD = ΔT_source_–ΔT_garden_, where ΔT is the difference between mean temperature of the warmest month minus mean temperature of the coldest month (*R* ^2^ = .95, *p* < .0001). Seedling survival was negatively related to CTD, as was variation in crown volume (cm^3^, *R* ^2^ = .55, *p* = .034), and proportion of reproducing individuals (*R* ^2^ = .92, *p* < .0001, Figure 3). The Kaplan–Meier survival analysis indicated no significant differences in the timing or extent of mortality between populations (*p* = .51), but mortality rates differed significantly between gardens (*p* < .0001, Figure 2).

**TABLE 3:** Type III ANOVA results assessing Garden and Population differences in the proportion of surviving individuals, crown volume (dm^3^), and the proportion of reproducing individuals

Factor	*df*	*F*	*p*
Survival			
Population	2	0.06	.95
Garden	2	456.03	**2.8 × 10^–7^**
Crown volume			
Population	2	1.133	.324
Garden	2	9.659	**.0001**
Garden × Population	2	0.83	.92
Reproduction			
Population	2	0.0442	.957
Garden	2	67.612	**7.7 × 10^–5^**

Note

Statistically significant effects (*p* < .05) are in bold.

**FIGURE 4:**
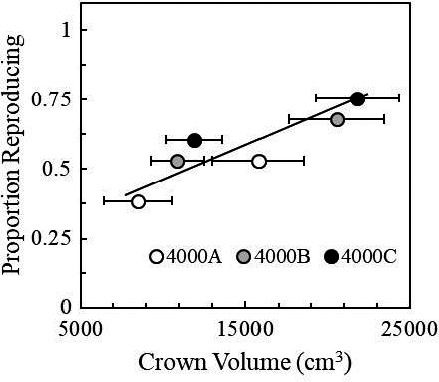
Proportion of reproducing individuals in relation to mean (±*SE*) crown volume (cm^3^). The proportion of reproducing individuals increases with increased crown volume (cm^3^, *R* ^2^ = .85, *p* = .002)

Reproductive effort was positively correlated with crown volume (*R* ^2^ = .85, *p* = .002, Figure 4). This relationship was further evaluated by quantifying the mean (±*SD*) number of reproductive stalks for 81 flowering individual big sagebrush growing in the Mid garden, where 34.8 (±27.6) reproductive stalks per plant were identified. The number of reproductive stalks per plant was also positively correlated to crown volume [(#stalks = 6^−05^ × (mm^3^ crown volume) + 6.5), *R* ^2^ = .45, *p* < .0001, T. Caughlin, unpublished data, fall 2018].

Seed source and garden locations occurred across four different soil taxonomic subgroups and two textures (Table 2). Seed source “A,” “C,” and the Mid garden occur on loam, Abruptic Xeric Argidurids. Seed source “B” occurs on loam, Xerollic Paleargids. The High garden occurs on loam, Argiduridic Durixerolls and the Low garden on sandy‐loam, Xeric Haplargids.

### 3.2 Seed zone delineation

Richardson and Chaney's (2018) empirical seed zone guidance identifies eight broadly defined zones across the entire Great Basin, and all three of our gardens and source populations occurred within the same seed zone (Zone 2). Bower et al.'s (2014) provisional seed‐transfer guidelines identify 64 climatic zones across the continental United States, with 34 existing within the Great Basin. Provisional seed zones are based upon 2.8°C bands of minimum winter temperatures from December through February combined with groupings of the annual heat: moisture index (AH:M) measure of aridity [mean annual temperature (°C) divided by mean annual precipitation (m)]. The three seed‐source sites and the middle garden (Mid) are all situated within the “−9.4 to −6.7°C/6–12 AH:M” provisional seed zone. The High and Low gardens were situated in the “−6.7 to −3.9°C/3–6” and “−6.7 to −3.9°C/6–12” provisional seed zones, respectively (Figure 1). Seedling survival was (a) greatest (99%) in the “−9.4 to −6.7°C/6–12 seed zone that the populations were native to, (b) least (2%) in the warmer “−6.7 to −3.9°C/6–12 seed zone, and (c) intermediate (72%) at the wetter High garden situated within the “−6.7 to −3.9°C/3–6 seed zone (Figure 2).

### 3.3 Weather during study relative to climate

**FIGURE 5:**
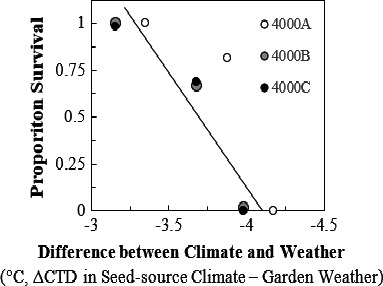
Proportion of surviving individuals relative to the difference between ΔCTD (based upon 30 year climate averages) and weather temperature difference (ΔWTD). Climatic Temperature Difference (°C, ΔCTD = ΔT_source_–ΔT_garden_) is calculated from 30‐year climate averages while Weather Temperature Difference (°C, ΔWTD = WTD_source_–WTD_garden_) is calculated from weather conditions experienced over the period of the study. Increasingly negative values of ΔCTD–ΔWTD indicate that temperature extremes were greater than climate averages would suggest and were correlated with reduced survival (*R* ^2^ = .76, *p* = .0024)

From November 2016 to April 2019, weather conditions at the gardens had lower minimum and greater maximum temperatures than climate averages. To evaluate whether survival patterns identified in relation to ΔCTD were consistent with the temperature difference experienced during the study period, the Weather Temperature Difference (WTD) was calculated in the same way as CTD but using the average annual difference between mean temperature of the warmest month and mean of the coldest month during the study period. Survival decreased significantly as mean annual temperature differences, specifically ΔCTD–ΔWTD (the difference between seed‐source climate and planting‐garden weather, during the study) became more negative (Figure 5). This indicated lower survival for seedlings placed into conditions of greater temperature extremes compared to temperature regimes of their site of population origin (*R* ^2^ = .76, *p* = .0024, Figure 5). Seedling mortality occurred in greatest proportions in July and August with the onset of increasing annual temperatures and decreasing precipitation (Figure 2) indicating that heat and drought stress may have contributed to observed mortalities, consistent with the climate factors used for provisional seed‐transfer zone delineation.

## 4 DISCUSSION

We detected a large difference in fitness metrics across a small spatial scale for all three populations alike, suggesting the specificity of their climate requirements can occur over a much finer grain than has been previously measured or considered within a subtaxa of big sagebrush or similar, widespread and wind‐pollinated species of semiarid regions. The differences in fitness that we observed was comparable or even greater than is typically observed for populations with greater distances between them (e.g., Brabec, Germino, & Richardson, 2017; Chaney, Richardson, & Germino, 2017; Germino, Moser, & Sands, 2019) and was also rapidly expressed within the relatively short 3 years of our study. Our findings contrast observations from the few other studies that evaluated the spatial scale of adaptive variation. Populations of wheat (*Triticum dicoccoides*, Volis, Ormanbekova, Yermekbayev, Song, & Shulgina, 2015) and an annual legume (*Chamaecrista fasciculata*, Galloway & Fenster, 2000) differed significantly in traits only when their origins were separated by large distances, such as >50 km or more, compared to within a few km. Similarly, no local adaptation was observed between populations of silver fir (*Abies alba*) from provenances ranging in geographic distance from 1.5 to 200 km (Latreille & Pichot, 2017) or between eight populations of common ash (*Fraxinus excelsior* L.) distributed across the species range in England and Wales (Boshier & Stewart, 2005). A “home‐site” advantage was observed between 11 populations of false dandelion (*Hypochaeris radicata*) collected from 3 to 548 km from the garden locations (Becker, Dostal, Jorritsma‐Wienk, & Matthies, 2008); however, examination of survival over their distances which are most comparable to our study (3 km and ~100 km) suggests no population differences. Evidence of local adaptation was strong in an arctic dwarf shrub (*Dryas octopetala*) and a tussock forming sedge (*Eriophorum vaginatum*); however, the population differences were much greater than 100 km (Bennington et al., 2012). The specificity of the three similar populations of big sagebrush for certain climate conditions detected here was also stronger than that detected in a previous study that evaluated population separation distances of ~50 to >700 km in the same species we evaluated, *A. t. wyomingesis,* in common gardens (Germino et al., 2019). The timeframe of the Germino et al. (2019) study was longer (>20 years) and we also measured climate translocation much more precisely.

Wyoming big sagebrush is the most broadly distributed subspecies within the *A. tridentata* complex and occurs across several ecoregions, thus a diverse array of drivers could contribute to adaptive variation among its populations (McKay et al., 2005; Rehfeldt, 1979). Genetic differentiation between geographically or environmentally isolated populations can result when differing conditions exert stronger selection than interpopulation gene flow can mitigate for. Big sagebrush pollen and seed are both wind‐dispersed, although its seeds are thought to have limited dispersal distances from the mother plants, less than a few meters. While pollen has the potential to travel long distances that could facilitate gene flow between populations, shifts in flower phenology between populations would result in genetic isolation and facilitate genetic differentiation (Richardson, Chaney, Shaw, & Still, 2017). Biotic selection pressures that could drive site‐specific adaptation may differ between garden sites, but the most substantial biotic differences among sites were relatively recent compared to the slow time course for microevolutionary diversification, such as cheatgrass invasion at the Low site in recent decades. Many herb species are common to all 3 sites; and, while insect herbivory could be a factor, we did not observe such impacts.

Spatial variation in climate is often a strong correlate of population differentiation at subspecific and ecotypic levels (Clewell & Rieger, 1997; Hufford, Mazer, & Camara, 2008; Liancourt et al., 2013) and is thus a primary basis for delineating seed‐transfer zones. Survival of big sagebrush in our common gardens ranged from 1% to 99%, and the common gardens all occurred in the same empirical seed zone among those delineated by Richardson and Chaney (2018). However, climate specificity of the three populations we observed corresponded well to Bower et al.'s (2014) provisional seed zones, which are based on climate information (and ecoregional identity for larger scale applications). The provisional seed zones are not parameterized with information about sagebrush specifically (i.e., with common‐garden data or other biological responses). One would expect that seed zones determined from empirical studies would better predict the population differences in survival that we observed, but it appears that the greater spatial and categorical resolution of the provisional seed zones were essential for predicting the differences in survival that we observed.

While adaptive variation in big sagebrush across broad, ecoregional scales is well known (Brabec et al., 2015; Richardson & Chaney, 2018), site‐specific or local adaptation at this scale can require decades to become evident (Germino et al., 2019). Broadscale adaptive variation in survival (Chaney et al., 2017) and flowering phenology (Richardson et al., 2017) were largely explained by climate‐of‐seed origin. There are fewer studies of adaptation to fine‐scale spatial conditions in big sagebrush, and the prior studies available focus on sharp gradients between subtaxa. Specifically, a reciprocal transplant study of big sagebrush subspecies *A. t*. *tridentata*, *A. t*. *vaseyana*, and their hybrid within and across their elevational zones on a slope in Utah revealed local adaptation within a narrow (1,200 m distance, 80 m in elevation) gradient within 2 years of planting (Wang, McArthur, Sanderson, Graham, & Freeman, 1997). The fine‐grained local adaptation reported by Wang et al. (1997) was attributable to morphologically (and, thus, taxonomically) distinctive hybrid formation. We report three populations within a single taxonomic subspecies (*A.t. wyomingensis*) that originated from similar site conditions had low or “no” fitness when moved to higher or lower elevations where the species otherwise appears to establish well and resident populations surrounding the gardens shared the same morphology and thus taxonomic identity of the planted populations.

Differences in subspecies and population tolerances and/or resistance to drought (Kolb & Sperry, 1999; McArthur et al., 1998) and cold temperature (Brabec et al., 2017; Chaney et al., 2017; Lambrecht, Shattuck, & Loik, 2007; Lazarus, Germino, & Richardson, 2019) have been linked to differences in survival of big sagebrush populations in common gardens. Soil properties, such as restrictive subsurface layers, affected survival of outplanted seedlings of big sagebrush (Davidson et al., 2019) and were important factors affecting adaptive variation of another widespread perennial of the western US (Gibson, Nelson, Rinehart, Archer, & Eramian, 2019). However, we did not observe evidence that restrictive subsurface layers or other soil factors contributed to the population differences in survival for this current study. Specifically, the “B” source population originated from a paleargid soil type, whereas populations “A” and “C” originated from abruptic argidurids that have near‐surface restrictive horizons (within 50 cm depth; Table 2). There were no differences in survival between these populations at any of the garden locations including at the Mid garden that had the same soil type as populations “A” and “C.” The argiduridic durixeroll soils of the High garden also have a restrictive layer, but it occurs much deeper in the soil profile (>100 cm depth) and thus these soils are more amenable to the deep‐soil growth requirements of big sagebrush (Germino & Reinhardt, 2014), and yet survival of all populations was less at this High garden compared to the Mid garden (Figure 3). Similarly, differences in soil texture were not likely to explain differences in survival of all the populations among the gardens, considering that all seed‐source and garden sites were classified as loam except for the sandy‐loam of the Low site where we would have expected greater infiltration to enhance sagebrush but instead experienced the least survival (Figure 3). In all, effects of the physical structure of the soil on seedling survival do not seem to be stronger than climatic selection.

### 4.1 Summary

The selection in our study was considerably strong, being evident in less than 3 years after planting and possibly reflecting the climate sensitivity of seedlings (Brabec et al., 2017; Germino et al., 2019). Our findings indicate that more resolution in empirical seed‐transfer zones would be necessary to capture the important population variability occurring within the study region. However, there are practical limits to the number of seed‐transfer zones that can be feasibly collected and used by and for land management (McKay et al., 2005), and so it seems a tradeoff between precision and practicality is unavoidable. At minimum, our findings offer a possible explanation for the lack of successful establishment following many historical seedings (Knutson et al., 2014). One temporary approach to satisfy the near‐term needs for seed‐transfer guidance would be to make a hybrid use of coarser empirical guidelines from Richardson and Chaney (2018) and the finer scale provisional guidelines from Bower et al. (2014), perhaps using the former for broadscale guidance for planning seed acquisition (which is typically a generalized activity not done with a specific seed destination in mind) and the latter to determine which of the collected seeds to apply to appropriate areas within specific restoration areas. Looking forward, the scale of local adaptation in big sagebrush and other species could be more precisely determined from new common gardens if they formally incorporated reciprocal plantings across varying distances from seed sources to gardens, from within meters to many kilometers (e.g., Galloway & Fenster, 2000). The resulting information could then be used to provide guidance on the appropriate spatial scale of seed‐transfer zone application.

## CONFLICT OF INTEREST

The authors state that there is no conflict of interest.

## AUTHOR CONTRIBUTIONS

**Bill E. Davidson:** Data curation‐Lead, Formal analysis‐Lead, Investigation‐Equal, Methodology‐Equal, Writing‐original draft‐Supporting. **Matthew J. Germino:** Conceptualization‐Lead, Formal analysis‐Supporting, Funding acquisition‐Lead, Methodology‐Equal, Project administration‐Lead, Resources‐Lead, Supervision‐Lead, Visualization‐Equal, Writing‐original draft‐Lead, Writing‐review & editing‐Lead.

## Data Availability

Data are deposited on sciencebase.org: https://doi.org/10.5066/P94FRKP6.
